# P-489. Opt-Out HIV Testing, Trends in a Community Healthcare System–A Longitudinal Analysis from 2018 to 2024

**DOI:** 10.1093/ofid/ofae631.688

**Published:** 2025-01-29

**Authors:** Paula A Eckardt, Jianli Niu, Jennifer L Rodriguez-Belen, Elizabeth Sherman, Sheila Montalvo, Elsa M Acevedo Martinez, Heysu Rubio-Gomez

**Affiliations:** Memorial Healthcare System, Hollywood, Florida; Memorial Healthcare System, Hollywood, Florida; Memorial Healthcare System, Hollywood, Florida; Memorial Healthcare Systems, Hollywood, Florida; Memorial Hospital System, Cooper City, Florida; Memorial Healthcare System, Hollywood, Florida; Memorial Healthcare System, Hollywood, Florida

## Abstract

**Background:**

Emergency department (ED)-based “Opt-Out” HIV testing is critical to access HIV prevention and care services. Due to the COVID-19 pandemic, disruptions in medical services might have affected HIV testing uptake.

The number of HIV tests per month in ED-based settings in the Memorial Healthcare System between July 2018 and February 2024. A, Pre-COVID-19 phase; B, The COVID-19 pandemic phase; C, Post-pandemic phase. MPC, monthly percent change

**Figure 1.**
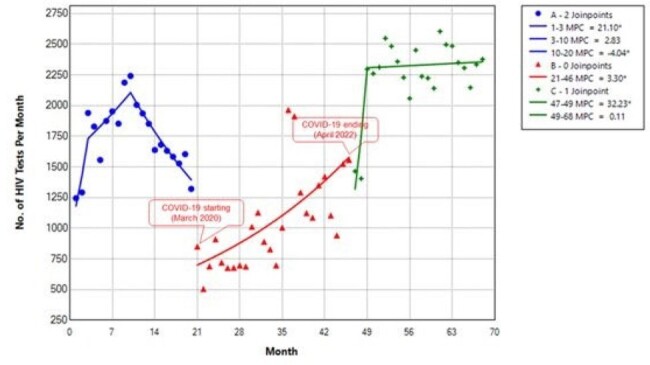
Multiple joinpoint models showing the temporal trends in the number of HIV tests per month in ED-based settings in the MHealthcare System between July 2018 and February 2024. A, Pre-COVID-19 phase; B, The COVID-19 pandemic phase; C, Post-pandemic phase. MPC, monthly percent change.

**Methods:**

We collected data on ED-based HIV testing from the Memorial Healthcare System in Hollywood, Florida. Data from July 2018 to February 2024 were divided into three phases: pre-pandemic (July 2018 – Feb 2020), pandemic (March 2020 − April 2022), and post-pandemic (May 2022 – February 2024) phases. Joinpoint regression models were used for analysis. The actual observed numbers of HIV testing that occurred from months beginning March 2020 to April 2022 were compared with the number of HIV tests predicted by the models.

Trends in positivity rates of HIV testing from July 2018 to February 2024, stratified by the COVID-19 pandemic.

**Figure 2.**
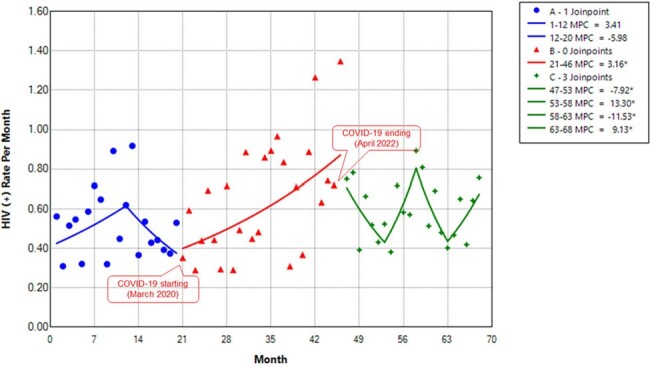
Multiple joinpoint models showing the temporal trends in the positivity rates of HIV tests per month in ED-based settings in the Memorial Healthcare System between July 2018 and February 2024. A, Pre-COVID-19 phase; B, The COVID-19 pandemic phase; C, Post-pandemic phase. MPC, monthly percent change

**Results:**

The monthly HIV tests remained stable during the pre-pandemic phase, with an average monthly percentage change (AMPC) of 0.88% (95% confidence interval [CI]: -0.38 to 1.98; p > 0.05). However, there was an increasing trend in monthly HIV tests during the pandemic phase, with an AMPC of 3.30% (95% CI, 2.98 to 4.49; p < 0.05).In the post-pandemic phase, monthly HIV tests remained increased, with an AMPC of 2.79% (95% CI, 1.86 to 4.22; p < 0.05). In the absence of the COVID-19 pandemic, the model projected 38,742 tests from March 2020 to April 2022 and 40,428 tests from May 2022 to February 2024. However, we observed 27,270 and 49,582 tests respectively, representing a 29.6% reduction for the pandemic phase (p < 0.001) and a 22.6% increase for the post-pandemic phase (p < 0.001).

**Figure:**
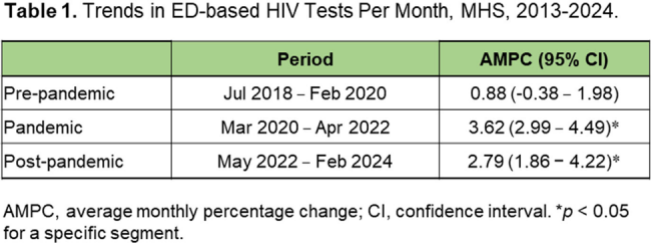

In the absence of the COVID-19 pandemic, the model projected 38,742 tests from March 2020 to April 2022 and 40,428 tests from May 2022 to February 2024. However, we observed 27,270 and 49,582 tests respectively, representing a 29.6% reduction for the pandemic phase (p < 0.001) and a 22.6% increase for the post-pandemic phase (p < 0.001).

**Conclusion:**

The number of HIV tests decreased substantially during the COVID-19 pandemic period. However, there was an increasing trend for HIV tests during the pandemic and the post-pandemic phases, with a MPCs of 3.30% and 2.79%, respectively.

Our findings correlate with other reports on decreased HIV diagnosis from 2019 to 2020. These findings have epidemiological significance and can inform health providers in implementing appropriate measures for HIV prevention.

In conformance to CDC recommendations, all health care systems should increase access to HIV testing services to mitigate the missed diagnoses during the COVID-19 pandemic, these strategies include self-testing and routine opt out screening in health care settings.

**Figure:**
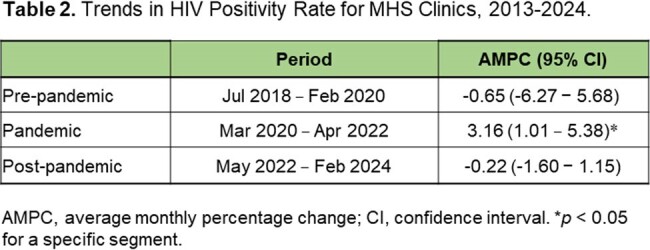

**Disclosures:**

**All Authors**: No reported disclosures

